# Extracellular vesicles derived from GMSCs stimulated with TNF-α and IFN-α promote M2 macrophage polarization via enhanced CD73 and CD5L expression

**DOI:** 10.1038/s41598-022-17692-0

**Published:** 2022-08-03

**Authors:** Yukari Watanabe, Takao Fukuda, Chikako Hayashi, Yuki Nakao, Masaaki Toyoda, Kentaro Kawakami, Takanori Shinjo, Misaki Iwashita, Hiroaki Yamato, Karen Yotsumoto, Takaharu Taketomi, Takeshi Uchiumi, Terukazu Sanui, Fusanori Nishimura

**Affiliations:** 1 Division of Oral Rehabilitation, Department of Periodontology, Faculty of Dental Science, Kyushu University, 3-1-1 Maidashi, Higashi-ku, Fukuoka, 812-8582 Japan; 2 Department of Dental and Oral Surgery, St. Mary’s Hospital, Fukuoka, Japan; 3 Department of Clinical Chemistry and Laboratory Medicine, Graduate School of Medical Sciences, Kyushu University, Fukuoka, Japan

**Keywords:** Cell biology, Molecular biology, Stem cells

## Abstract

Immunoregulatory properties of mesenchymal stem cell (MSC)-derived extracellular vesicles (EVs) are promising. Gingival tissue-derived MSCs (GMSCs) have unique immunoregulatory capacity and secrete large amounts of EVs. Recent findings suggest that priming MSCs with inflammatory stimuli is an effective strategy for cell-free therapy. However, the precise mechanism by which the contents of EVs are customized has not been fully elucidated. Here, we show that EVs derived from GMSCs primed with a combination of two pro-inflammatory cytokines, tumor necrosis factor-α (TNF-α) and interferon-α (IFN-α), synergistically promote anti-inflammatory M2 macrophage polarization by increasing the expression of cluster of differentiation 73 (CD73) and CD5 molecule-like (CD5L). Expression of CD73 by TNF-α/IFN-α stimulation was transcriptionally upregulated by the activation of mammalian target of rapamycin signaling and nuclear translocation of hypoxia-inducible factor 1α in GMSCs. TNF-α/IFN-α treatment also significantly increased the expression of CD5L mRNA via the transcription factor DNA-binding protein inhibitor ID3 and liver X receptor. Interestingly, exosomal CD5L is a prerequisite for the synergistic effect of EVs-mediated M2 macrophage polarization. These results indicate that combined pre-licensing with TNF-α and IFN-α in GMSCs is ideal for enhancing the anti-inflammatory function of EVs, which contributes to the establishment of a therapeutic tool.

## Introduction

Mesenchymal stem cells (MSCs) have been used for the treatment of a wide range of autoimmune disorders and inflammatory diseases because of their strong immunosuppressive and anti-inflammatory effects^1^. It was proposed that both transplanted MSCs and their conditioned media were able to exert therapeutic effects and that the paracrine mediators of MSCs were essential for immunoregulation^2^. The trophic secretome of MSCs includes soluble factors, such as transforming growth factor-β (TGF-β), IL-10, prostaglandin E2 (PGE2), indoleamine 2,3-dioxygenase (IDO), and tumor necrosis factor α (TNFα)-stimulated gene protein-6, which contribute to MSC-mediated immunosuppression^3,4^. In addition to these cytokines and growth factors, emerging evidence has demonstrated that MSC-derived extracellular vesicles (EVs) also contribute to the therapeutic effects of MSCs^5^. MSC-derived EVs have attracted great attention for cell-free MSC therapy because of their ability to transport vesicular cargo molecules, including cytokines, growth factors, and regulatory miRNAs, which mediate cell-to-cell communication^6^.

Accumulating evidence has demonstrated that MSCs mediate immunomodulatory effects by targeting the innate and adaptive immune cells via the secretion of immunosuppressive molecules when exposed to an inflammatory environment^1^. Among the innate immune cells, macrophages play central roles in the first line of host defense, both during the onset and resolution of inflammation. Macrophages are broadly classified into two phenotypes, pro-inflammatory M1 and wound-healing M2 cells, which correlate with the T-helper 1 and T-helper 2 (Th1/Th2) nomenclature^7^. Classically activated M1 macrophages are induced by Th1 cytokines, such as interferon-γ (IFN-γ) or lipopolysaccharide (LPS), and trigger acute inflammation by producing inflammatory cytokines, such as interleukin-1β (IL-1β), TNF-α, and IL-6. In contrast, alternatively activated M2 macrophages are induced by IL-4 and IL-13 through the activation of the common IL-4 receptor α signaling and production of anti-inflammatory cytokines such as IL-10, TGF-β, and VEGF^8^. More recently, adenosine signaling has been reported to be involved in macrophage polarization^9^. Adenosine is a powerful immunosuppressive mediator and its production is regulated by CD39 and CD73, which coordinately catalyze the hydrolysis of inflammatory adenosine triphosphate to generate adenosine. Adenosine-A_2A_ receptor (R)-mediated signaling suppresses inflammatory M1 macrophage activity^10,11^, whereas adenosine signaling via A_2B_R converts the phenotypic switch from M1 to M2 macrophages^12^. Specifically, MSC-derived EVs play a pivotal role in the crosstalk between MSCs and macrophages to enhance M2 macrophage polarization while suppressing M1 macrophage polarization^13^. Since M2 macrophages contribute to the resolution of inflammation and the subsequent tissue-remodeling process^14^, M2 macrophage induction by MSC-derived EVs is a novel therapeutic strategy for a wide range of diseases^15^, including osteolytic inflammatory diseases, such as periodontitis^16^. Furthermore, recent studies have indicated that MSCs can be activated by disease-related inflammatory signals and can increase their trophic effects by optimizing the contents of EVs to efficiently support the repair of specific diseases^17^.

Stem cells of dental origin have advantages of easy acquisition by a routine minimal invasion and superior potential in regenerative therapeutics^18^. Among the dental stem cells, gingival tissue-derived MSCs (GMSCs) are characterized by their prominent immunomodulatory and proliferative capacities that do not diminish at higher passage numbers^19^. Importantly, GMSCs secrete large amounts of EVs than those secreted by other somatic MSCs. TNF-α preconditioning further enhances the release of IL-1RA-containing EVs^20^. In our previous study, we compared the effect of preconditioning in GMSCs using LPS, TNF-α, IFN-γ, and acetylsalicylic acid and observed that TNF-α-stimulated GMSC-derived EVs significantly enhanced M2 activation, thereby exerting therapeutic effects in both wound healing and periodontal disease mouse models^16^. Based on the evidence that adenosine signaling is involved in macrophage phenotype^9^, we further revealed that TNF-α-enhanced exosomal CD73 expression was essential for inducing anti-inflammatory M2 macrophage polarization^16^. MSC preconditioning is an important tool for improving the effects of MSCs. It has been reported that cytokine stimulation affects the immunophenotype of MSCs by targeting Toll-like receptor (TLR)^21^ and combined priming of TNF-α and IFN-γ has demonstrated a synergistic effect^22,23^, suggesting that combination preconditioning of MSCs could be a novel strategy to improve the anti-inflammatory properties of MSC-derived EVs.

Although cluster of differentiation 73 (CD73) is a well-characterized MSC marker, it is abundantly expressed in vascular endothelial cells. Expression of CD73 in endothelial cells is induced by IFN-α, but not by lymphocytes^24^, indicating that induction of CD73 by IFN-α depends on the cell type. IFN-α enhances TLR3-mediated antiviral activity by inducing protein kinase R (PKR)^25^. We previously reported that PKR in the dental pulp cell-derived microvesicles is critical as a powerful inducer of inflammation^26^. However, to our knowledge, no study has been published regarding the effects of IFN-α on MSC priming, and the effect of IFN-α in combination priming with TNF-α on the negative feedback loop of GMSCs remains unclear. Therefore, in this study, we examined the effect of dual stimulation of TNF-α and IFN-α on GMSC-derived EVs-mediated M2 macrophage polarization and explored the detailed molecular signaling.

## Results

### Combined pre-conditioning of GMSCs with TNF-α and INF-α promoted CD73 expression in EVs

**Figure 1:**
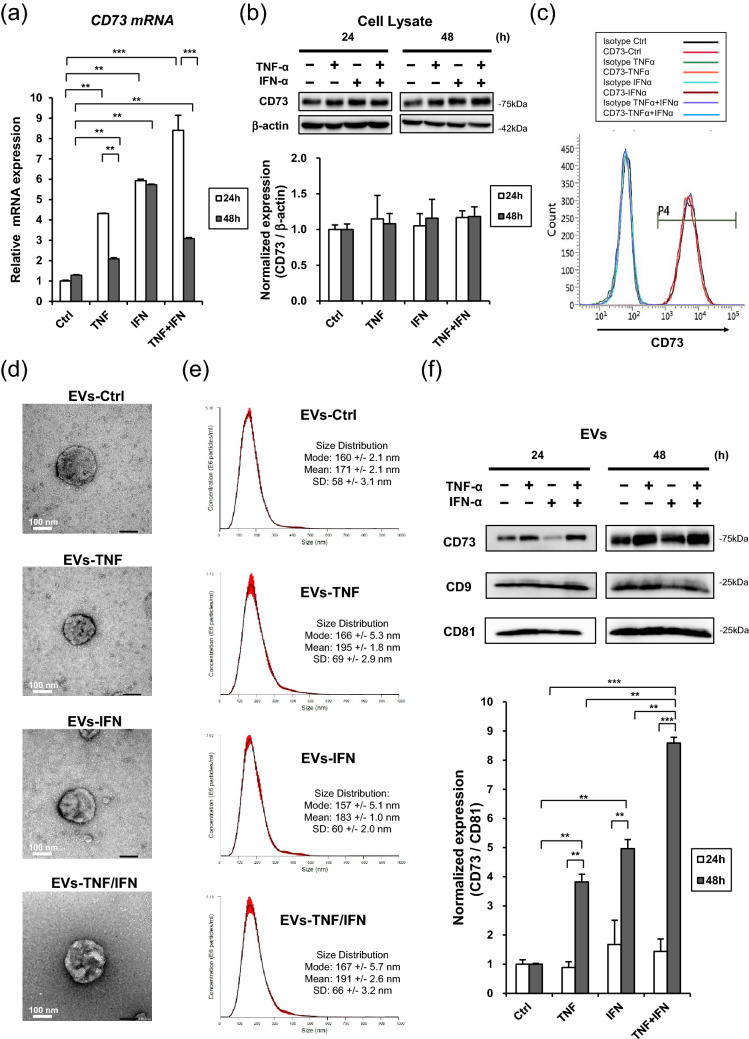
Effect of TNF-α/IFN-α stimulation on EVs and cellular expression of CD73 in GMSCs. GMSCs were stimulated with TNF-α (100 ng/mL), IFN-α (100 ng/mL), and TNF-α/IFN-α (50 ng/mL each) for 24 and 48 h. (**a**) Expression of CD73 mRNA was determined by quantitative real-time PCR. *GAPDH* was used as an internal control. (**b**) Whole-cell lysates prepared from GMSCs were subjected to western blot analysis using anti-CD73 antibody. β-Actin was used as a loading control for the cytosolic proteins (upper). Relative CD73 protein expressions were measured by quantifying the density of bands and normalized against β-actin using the MultiGauge software (lower). (**c**) Cell surface expression of CD73 was evaluated by flow cytometry. (**d**) Representative transmission electron micrographs (TEM) of EVs from GMSC with or without (EVs-Ctrl) priming of TNF-α (EVs-TNF), IFN-α (EVs-IFN), and TNF-α/IFN-α (EVs-TNF/IFN). Scale bar = 100 nm. (**e**) Size distribution of EVs-Ctrl, EVs-TNF, EVs-IFN and EVs-TNF/IFN were measured by NanoSight analyzer. (**f**) CD73 protein expression in EVs was compared in equal amounts of protein (5 μg) (upper). Exosomal positive marker of CD9 and CD81 proteins were used as loading controls. Relative CD73 protein expressions were measured by quantifying the density of bands and normalized against CD81 using the MultiGauge software (lower). **P < 0.01; ***P < 0.001. Error bars represent mean ± SD, *n* = 3. The significance of differences between groups was determined using one-way Tukey's test. Full blots are presented in Supplementary materials.

To examine the effect of TNF-α and IFN-α on the expression of CD73, GMSCs were stimulated with 100 ng/mL of TNF-α and INF-α, and 50 ng/mL TNF-α/IFN-α for 48 h and GMSC-derived EVs were isolated in accordance with our established protocol^16^. Quantitative real-time PCR (qRT-PCR) demonstrated that the combination of TNF-α and IFN-α synergistically increased the expression of CD73 mRNA after 24 h of stimulation, which was attenuated after 48 h, except for IFN-α stimulation (Fig. 1a). However, western blot analysis revealed that the protein expression levels of cellular CD73 in GMSCs remained the same after TNF-α, IFN-α, and TNF-α/IFN-α stimulation (Fig. 1b). Similarly, flow cytometry analysis demonstrated that the cell surface expression of CD73, a well-accepted MSC positive marker, was not affected in response to these cytokine stimuli (Fig. 1c). The EVs from GMSC with or without (EVs-Ctrl) priming of TNF-α (EVs-TNF), IFN-α (EVs-IFN), and TNF-α/IFN-α (EVs-TNF/IFN) displayed classic type of spherical morphology under the transmission electron microscopy (TEM) (Fig. 1d). Nanoparticle tracking analysis (NTA) further confirmed the purity and size distribution of the EVs. The most abundant particle sizes of EVs-Ctrl, EVs-TNF, EVs-IFN and EVs-TNF/IFN were almost the same (Mode: 160 ± 2.1 nm, 166 ± 5.3 nm, 157 ± 5.1 nm and167 ± 5.7 nm, respectively) (Fig. 1e). However, CD73 expression in EVs was significantly increased after the treatment of GMSCs with TNF-α and TNF-α/IFN-α (Fig. 1f). These results indicate that TNF-α and TNF-α/IFN-α-induced CD73 proteins were released by CD73-overexpressed EVs from GMSCs.

### TNF-α and INF-α-induced CD73 expression in GMSCs were mediated by mammalian target of rapamycin (mTOR)-hypoxia-inducible factor-1α (HIF-1α) axis

**Figure 2:**
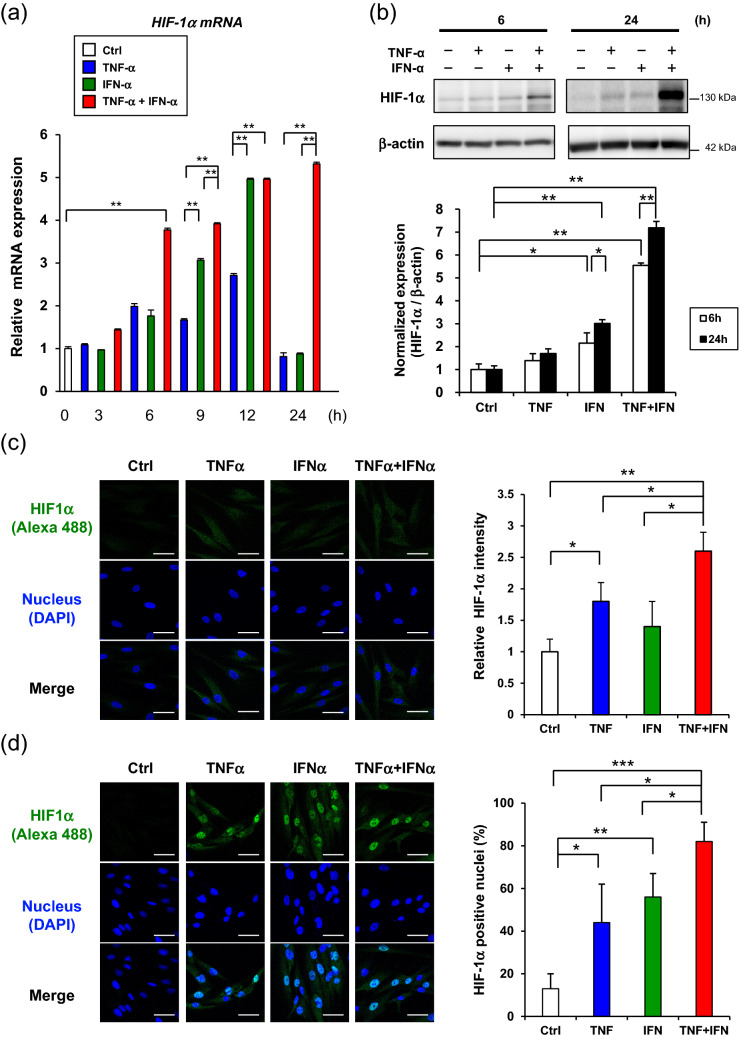
TNF-α/IFN-α stimulation accumulates nuclear expression of HIF-1α in GMSCs. (**a**) Time-course expression of HIF-1α mRNA in TNF-α/IFN-α-stimulated GMSCs. *GAPDH* was used as an internal control. (**b**) After stimulation by TNF-α/IFN-α for 6 h and 24 h, whole-cell lysates prepared from GMSCs were subjected to immunoblot analysis with an anti-HIF-1α antibody. β-actin was used as a control. Representative western blot analysis of total Grp78 is shown in the upper panel. Quantification of total HIF-1α protein levels relative to β-actin was performed using MultiGauge software (lower panel). (**c**,**d**) Time course confocal images of HIF-1α internalization. GMSCs were incubated with TNF-α/IFN-α for 6 h (**c**) and 24 h (**d**), respectively. The cells were stained with anti-HIF-1α antibody followed by an Alexa 488 secondary antibody (green). Nuclei were stained with DAPI dye (blue). The intensities of HIF-1α fluorescence (**c**; right) and nuclear translocated HIF-1α (**d**; right) for each sample were measured from all the cells in each group, which were counted in three randomly chosen fields. Scale bars: 20 μm. *P < 0.05; **P < 0.01; ***P < 0.001. Error bars represent mean ± SD, *n* = 3. The significance of differences between groups was determined using one-way Tukey's test. Full blots are presented in Supplementary materials.

**Figure 3:**
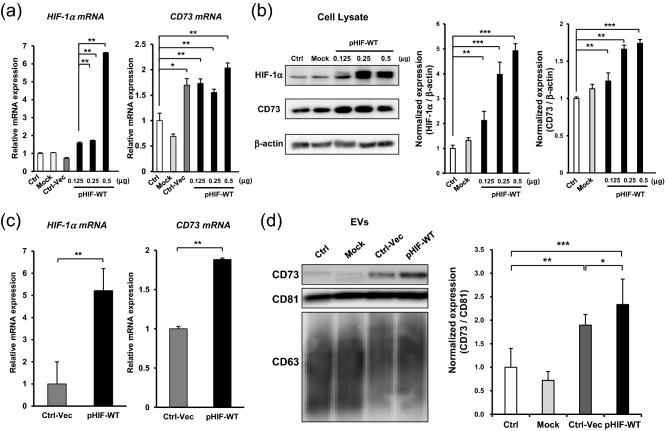
HIF-α regulates the expression of CD73 in GMSCs. (**a**,**b**) Effect of HIF-1α-overexpression on cellular CD73 expression in GMSCs. GMSCs were seeded on 6-well plates (5 × 10^5^ cells/well) a day before transfection. At approximately 60–70% confluence, the cells were transfected with indicated amounts of HIF-1α-overexpressed plasmid (pHIF-WT) (0.125, 0.25, and 0.5 μg/2 mL) for 24 h. The mRNA and protein expression of HIF-1a and CD73 in GMSC was analyzed by quantitative real-time PCR (**a**) and western blot analysis (**b**). Relative HIF-1α and CD73 protein expressions were measured by quantifying the density of bands and normalized against β-actin using the MultiGauge software (right). After transfection of pHIF-WT (0.25 µg/2 mL) for 48 h (**c**), GMSC-derived EVs were isolated from culture media, and the expression of CD73 in EVs was evaluated by western blotting (**d**). Exosome-associated CD63 and CD81 proteins were used as loading controls. Relative CD73 protein expressions were measured by quantifying the density of bands and normalized against CD81 using the MultiGauge software (**d**; right). *P < 0.05; **P < 0.01; ***P < 0.001. Error bars are means ± SD., *n* = 3. Data represent mean ± SD. The significance of differences between groups was determined by one-way Tukey's test. Full blots are presented in Supplementary materials.

**Figure 4:**
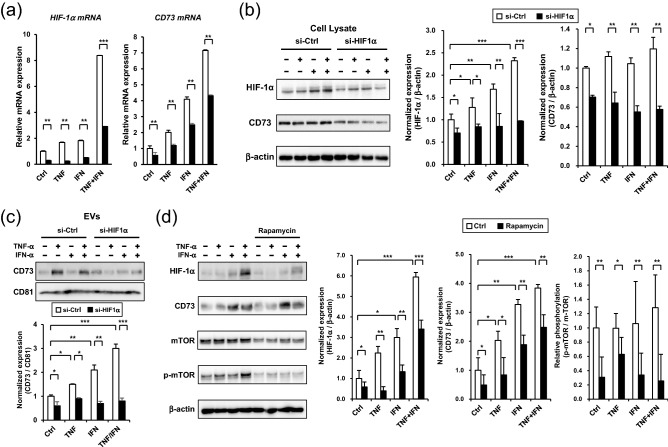
mTOR-HIF-α axis is critical for TNF-α/IFN-α-mediated induction of CD73 in GMSCs. GMSCs transfected with either control (si-Ctrl) or HIF-1α siRNA (si-HIF1α) were stimulated with TNF-α (100 ng/mL), IFN-α (100 ng/mL), or TNF-α/IFN-α (50 ng/mL) for 24 h (**a**) and 48 h (**b**–**d**). (**a**) Total RNA was isolated and analyzed to evaluate HIF-1α and CD73 mRNA expression using quantitative real-time PCR. (**b**) Whole-cell lysates prepared from GMSCs were subjected to immunoblot analysis with anti-CD73 or anti-HIF-1α antibodies. β-actin was used as the control. Relative HIF-1α and CD73 protein expressions were measured by quantifying the density of bands and normalized against β-actin using the MultiGauge software (right). (**c**) Exosomal CD73 protein expression in equal amounts (5 μg) was compared by immunoblot analysis with an anti-CD73 antibody. Exosome-associated CD81 proteins were used as loading controls. Relative CD73 protein expressions were measured by quantifying the density of bands and normalized against CD81 using the MultiGauge software (lower). (**d**) Effect of rapamycin treatment on the expression of HIF-1α and CD73 in TNF-α, INF-α, and TNF-α/ INF-α-stimulated GMSCs. Inhibition of mammalian target of rapamycin (mTOR) activity was confirmed using a phosphorylated-mTOR antibody (p-mTOR, Se2448). Relative HIF-1α and CD73 protein expressions were measured by quantifying the density of bands and normalized against β-actin, and relative phosphorylation of mTOR were measured by quantifying the density of bands and normalized against mTOR using the MultiGauge software (right). using the MultiGauge software (**b**; right). *P < 0.05; **P < 0.01; ***P < 0.001. Error bars represent mean ± SD, *n* = 3. The significance of differences between groups was determined using one-way Tukey's test. Full blots are presented in Supplementary materials.

Further, we explored the universal molecular mechanisms of TNF-α and IFN-α-mediated expression of CD73 in GMSCs. Considering the recent findings of the induction of HIF-1α by inflammatory cytokines^27^ and the regulation of CD73 expression by HIF-1α, we hypothesized that the TNF-α/IFN-α-mediated upregulation of CD73 could be induced by HIF-1α. To test this hypothesis, we first confirmed that dual TNF-α and IFN-α stimulation synergistically enhanced the expression of HIF-1α mRNA in GMSCs (Fig. 2a). Western blot analysis further confirmed the dual stimulation-induced upregulation of HIF-1α protein expression for 24 h (Fig. 2b). Immunofluorescent staining captured by confocal microscopy demonstrated that both TNF-α and IFN-α-induced the expression of HIF-1α within 6 h (Fig. 2c). Furthermore, significant nuclear accumulation of HIF-1α, which indicates the activation of HIF-1α, was observed after GMSCs were treated with TNF-α and IFN-α for 24 h (Fig. 2d). To examine the direct involvement of HIF-1α in TNF-α and IFN-α-mediated upregulation of CD73 expression, GMSCs were transfected with a HIF-1α overexpression plasmid (pHIF1) or siRNA for HIF-1α (siHIF1). Twelve hours after transfection, pHIF1 enhanced HIF-1α mRNA expression in GMSCs in a dose-dependent manner. However, the expression levels of CD73 mRNA were unchanged (Fig. 3a). However, increased expression of CD73 and HIF-1α was observed 24 h after transfection with pHIF1 (Fig. 3b), suggesting that the expression of CD73 was induced after enhanced expression of HIF-1α in GMSCs. In accordance with our established protocol, EVs from pHIF1 transfected GMSCs were isolated from cell culture supernatants. Forty-eight hours after transfection, the expression levels of both HIF-1α and CD73 mRNA were still elevated (Fig. 3c). Similarly, a significant increase in the exosomal CD73 expression was observed in pHIF1-transfected GMSCs compared to that in control transfectants (Fig. 3d). In contrast, transfection with siHIF1 attenuated both the TNF-α and IFN-α-induced expression of CD73 mRNA (Fig. 4a) and endogenous expression levels of CD73 protein in GMSCs (Fig. 4b). Consequently, the knockdown of HIF-1α suppressed the expression of CD73 in EVs from GMSCs (Fig. 4c). We further examined the pathway involved in HIF-1α-mediated CD73 expression. As HIF-1α expression is known to be regulated by the mTOR pathway^27^, rapamycin was used to inhibit mTOR. mTOR inhibition decreased both HIF-1a and CD73 expression in response to TNF-α/IFN-α stimuli (Fig. 4d). These results suggest that TNF-α and INF-α-induced CD73 expression is regulated by the activation of mTOR signaling and the subsequent nuclear translocation of HIF-1α in GMSCs.

### EVs from TNF-α and INF-α-stimulated GMSCs synergistically reprogram macrophage from M1 to M2 phenotype

**Figure 5:**
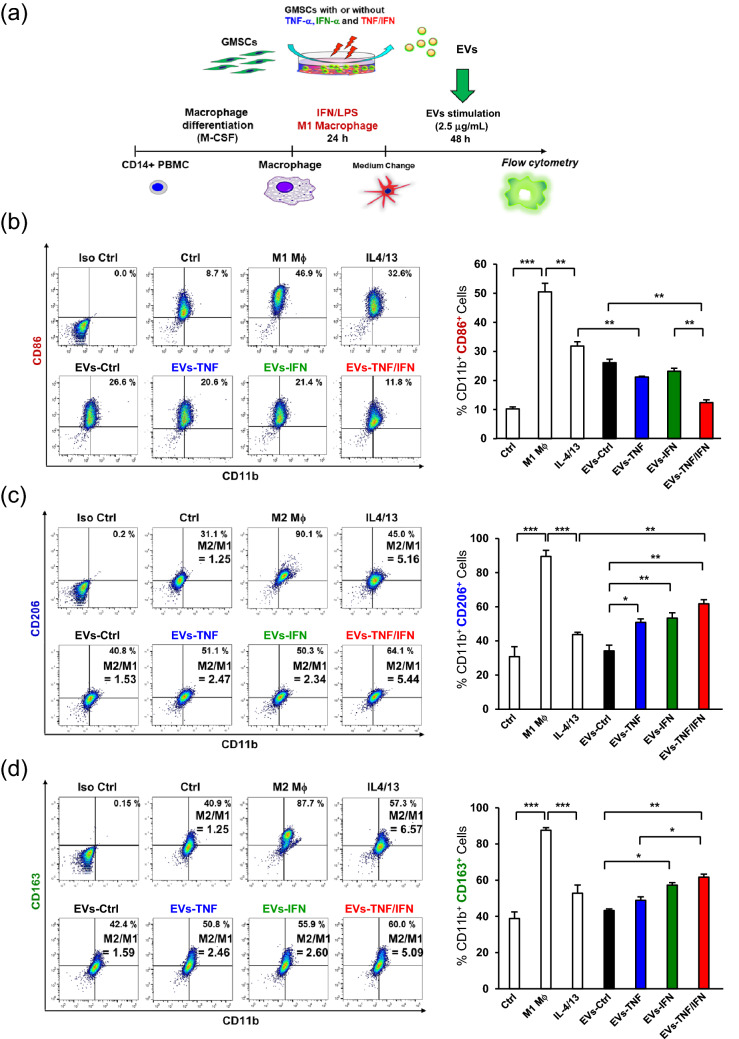
EVs from TNF-α/IFN-α-stimulated GMSCs enhance M2 macrophage polarization. (**a**) Strategy diagram for the validation of phenotypic switching capacity of EVs from M1 to M2 macrophages. After M1 macrophages were generated by LPS (10 ng/mL) and IFN-γ (25 ng/mL) stimulation from resting macrophages derived from CD14^+^ peripheral blood-derived monocytes (PBMCs) for 24 h, EVs (2.5 µg/mL) from GMSC with or without (Exo-Ctrl) priming of TNF-α (100 ng/mL; Exo-TNF), IFN-α (100 ng/mL; Exo-IFN), and TNF-α/IFN-α (50 ng/mL, each; Exo-TNF/IFN) were incubated with M1 macrophages for 48 h. (**b**) Effects of cytokine primed GMSCs-derived EVs on CD86 expression in M1 macrophages. (**c**,**d**) Effects of TNF-α-treated GMSC-derived EVs on CD206 (c) or CD163 (**d**) expression in M1 macrophages. Percentage of double positive cells (CD11b^+^ CD206^+^) (**c**) and (CD11b^+^ CD163^+^) (**d**) was analyzed to measure the ratio of macrophages polarized to M2 phenotype. Percentage of double positive cells (CD11b^+^ CD86^+^) representing M1 macrophages (**b**), and M2/M1 balance was determined by using the ratio of (CD11b^+^ CD206^+^)/(CD11b^+^ CD86^+^) (**c**) or (CD11b^+^ CD163^+^)/(CD11b^+^ CD86^+^) (**d**) macrophage population. *P < 0.05; **P < 0.01; ***P < 0.001. Error bars represent mean ± SD, *n* = 3. The significance of differences between groups was determined using one-way Tukey's test.

Our previous study confirmed that EVs from TNF-α-stimulated GMSCs enhanced the ability to convert the macrophage phenotype from pro-inflammatory M1 to anti-inflammatory M2 and further demonstrated that the expression level of CD73 in EVs was associated with the ability of EVs to switch M1 macrophages into M2 phenotype^16^. To compare and validate the modulatory effect of TNF-α and IFN-α preconditioning on macrophage reprogramming from the M1 to M2 phenotype, GMSCs were stimulated with TNF-α and INF-α individually and in combination before isolation of EVs from the cell culture supernatant. To achieve this, M1-activated macrophages were stimulated with or without EVs, and the expression of M1 (CD86) and M2 markers (CD206 and CD163) were examined by flow cytometry (Fig. 5a). Compared with GMSC-derived EVs without preconditioning (EVs-Ctrl), stimulation with TNF-α-preconditioned GMSC-derived EVs (EVs-TNF) showed a decreased expression of CD86 (26.6% vs. 20.6%) (Fig. 5b) and an enhanced expression of CD206 and CD163 (40.8% vs. 51.1% and 42.4% vs. 50.8%, respectively) (Fig. 5c,d). While the effect of IFN-α-preconditioned GMSC-derived EVs (EVs-IFN) on the expression of M1/M2 markers was almost similar to that of EVs-TNF, TNF-α-, and IFN-α-preconditioned GMSC-derived EVs (EVs-TNF/IFN), demonstrating a significant reduction in M1 marker expression (CD86: 11.8%) (Fig. 5b) and increase in M2 marker expression (CD206: 64.1%, CD163: 60.0%) (Fig. 5c,d). Consequently, EVs-IFN dramatically upregulated the M2/M1 ratio by approximately twofold relative to that of EVs-TNF (CD206/CD86: 2.47 vs. 5.44, and CD163/CD86: 2.46 vs. 5.09), and it was almost equal to IL4/13 stimulation after M1-activation (CD206/CD86: 5.16 vs. 5.44 and CD163/CD86: 6.57 vs. 5.09) (Fig. 5c,d). These results indicated that the combined preconditioning of GMSCs with TNF-α and IFN-α synergistically augmented the effect of EVs-mediated M2 macrophage reprogramming.

### TNF-α and INF-α stimulation induced CD5L expression in GMSCs-derived EVs contributed M2 macrophage polarization

**Figure 6:**
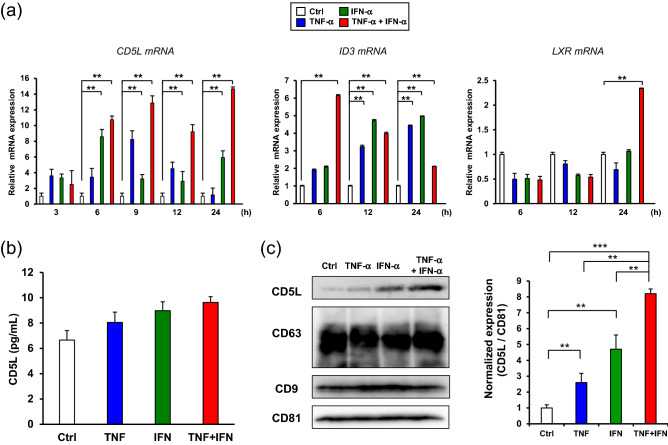
TNF-α/IFN-α stimulation increased CD5L expression in GMSCs-derived EVs. (**a**) Time-course expression of CD5L, ID3, and LXR mRNA in TNF-α (100 ng/mL), IFN-α (100 ng/mL), and TNF-α/IFN-α (50 ng/mL, each)-stimulated GMSCs. *GAPDH* was used as an internal control. (**b**) Expression of CD5L protein in the cell culture supernatants from cytokine-stimulated GMSCs for 48 h were quantified by ELISA. (**c**) Exosomal CD5L protein expression in equal amounts of the protein (5 μg) was compared by immunoblot analysis with an anti-CD5L antibody. Exosome-associated CD63, CD9, and CD81 proteins were used as loading controls. Relative CD5L protein expressions were measured by quantifying the density of bands and normalized against CD81 using the MultiGauge software (right). **P < 0.01; ***P < 0.001. Error bars represent mean ± SD, *n* = 3. The significance of differences between groups was determined using one-way Tukey's test. Full blots are presented in Supplementary materials.

**Figure 7:**
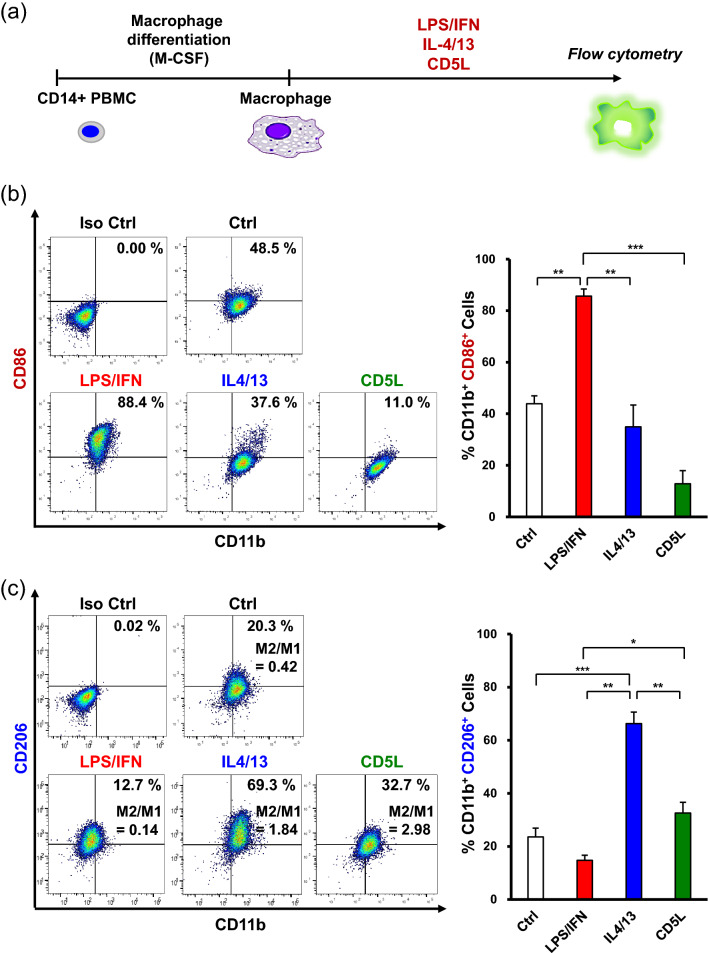
Recombinant CD5L enhanced M2 macrophage polarization. (**a**) Experimental diagram for CD14^+^ peripheral blood-derived monocytes (PBMC)-differentiated macrophage stimulation using recombinant human CD5L protein. After monocytes differentiate macrophages, cells were treated with LPS (10 ng/mL) plus of IFN-γ (25 ng/mL) (LPS/IFN), IL-4 plus IL-13 (25 ng/mL, each; IL-4/13) or of recombinant CD5L (1 μg/mL) for 72 h, respectively. (**b**) Effects of CD5L stimulation on CD86 expression in macrophages. (**c**) Percentage of double positive cells (CD11b^+^ CD206^+^) was analyzed to measure the ratio of macrophages polarized to M2 phenotype and M2/M1 balance was determined by using the ratio of (CD11b^+^ CD206^+^)/(CD11b^+^ CD86^+^) macrophage population. *P < 0.05; **P < 0.01; ***P < 0.001. Error bars represent mean ± SD, *n* = 3. The significance of differences between groups was determined using one-way Tukey's test.

**Figure 8:**
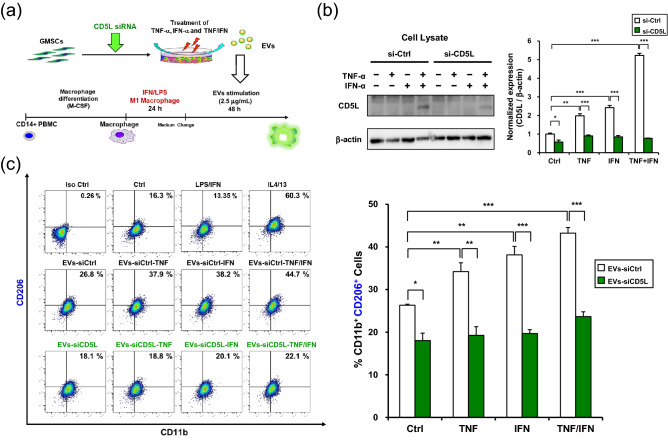
Effect of CD5L knockdown on TNF-α/IFN-α-stimulated GMSC-derived EVs -mediated M2 macrophage polarization. (**a**) Strategy diagram for the validation of phenotypic switching capacity of EVs from M1 to M2 macrophages. GMSCs were transfected with control (si-Ctrl) or CD5L siRNA (si-CD5L) for 48 h. EVs (2.5 µg/mL) from GMSCs transfected with either control (EVs-siCtrl) or CD5L siRNA (EVs-siCD5L) and subsequently stimulated with TNF-α (100 ng/mL; -TNF), IFN-α (100 ng/mL; -IFN), and TNF-α/IFN-α (50 ng/mL, each; -TNF/IFN) for 48 h were purified from each culture medium. After M1 macrophages were generated by LPS (10 ng/mL) and IFN-γ (25 ng/mL) stimulation from resting macrophages derived from CD14^+^ peripheral blood-derived monocytes (PBMCs), the GMSC-derived EVs were incubated with M1 macrophages for 48 h. (**b**) Whole-cell lysates of GMSCs prepared from culture conditions were subjected to immunoblot analysis with anti-CD5L antibody (left). β-actin was used as the control. Relative CD5L protein expressions were measured by quantifying the density of bands and normalized against β-actin using the MultiGauge software (right). (**c**) Effects of CD5L-siRNA-transfected GMSC-derived EVs on CD206 expression in macrophages. Percentage of double positive cells (CD11b^+^ CD206^+^) was analyzed to measure the ratio of macrophages polarized to M2 phenotype. *P < 0.05; **P < 0.01; ***P < 0.001. Error bars represent mean ± SD, *n* = 3. The significance of differences between groups was determined using one-way Tukey's test. Full blots are presented in Supplementary materials.

To gain further insights into the EVs-TNF/IFN-mediated M2 macrophage polarization, we hypothesized that the exosomal proteome of immunomodulatory proteins could contribute to the synergistic effect of TNF-α and INF-α stimulation in GMSCs. Since recent studies have demonstrated that CD5 molecule-like (CD5L)/apoptosis inhibitor of macrophages is expressed in human bone marrow MSCs (hBM-MSCs)^28^ and is involved in M2 macrophage polarization^29^, we examined the effects of TNF-α/IFN-α stimulation on the expression of CD5L mRNA in GMSCs (Fig. 6a). Combined stimulation with TNF-α and INF-α dramatically upregulated the expression of CD5L mRNA in comparison with TNF-α or IFN-α stimulation after 24 h. To validate if this effect was transcriptionally controlled by the DNA-binding protein inhibitor ID3 (ID3)^29^ and liver X receptor (LXR)^30^, time-course expression was monitored. Dual stimulation of TNF-α and INF-α significantly upregulated ID3 mRNA within 6 h and gradually decreased within 24 h. However, induction of LXR mRNA peaked at 24 h. Although the amount of secreted CD5L by TNF-α/IFN-α stimulation was less than 10 pg/mL and the stimulation had little effect on secretion for 48 h (Fig. 6b). TNF-α/IFN-α stimulation clearly enhanced CD5L expression in GMSC-derived EVs (Fig. 6c). To examine whether CD5L could induce M2 macrophage polarization, the macrophages were stimulated with recombinant CD5L (Fig. 7a). CD5L strongly decreased M1 marker expression lower than the IL-4/13 stimulation (37.6% vs. 11.0%) (Fig. 7b) and enhanced M2 marker expression relative to the unstimulated control macrophages (20.3% vs. 32.7%) (Fig. 7b). To further explore the role of exosomal CD5L in M2 macrophage polarization, GMSCs were transfected with CD5L siRNA before preconditioning and M1 polarized macrophages were treated with EVs (Fig. 8a). The efficiency of the CD5L knockdown (si-CD5L) and the upregulation of CD5L by TNF-α/INF-α stimulation in control siRNA (si-Ctrl) transfected GMSCs were confirmed by western blot analysis (Fig. 8b). Compared to unstimulated control (Ctrl), control siRNA-transfected GMSC-derived EVs (EVs-siCtrl) upregulated CD206 expression (16.3% vs. 26.8%). which was further enhanced by dual stimulation of GMSCs with TNF-α and INF-α (EVs-siCtrl-TNF/IFN) (26.8% vs. 44.7%; 1.67-fold). Conversely, CD206 expression was decreased by knockdown of CD5L in GMSC-derived EVs (EVs-siCD5L) (26.8% vs. 18.1%) and the effect of M2 activation by TNF-α and INF-α stimulation (EVs-siCD5L-TNF/IFN) was attenuated (18.1% vs. 22.1%; 1.22-fold) relative to the siRNA controls (Fig. 8c). These results suggested that TNF-α and INF-α stimulation-induced CD5L in GMSC-derived EVs also contributed to M2 macrophage activation.

## Discussion

This study aimed to establish an effective and reproducible protocol for GMSC-derived EVs-based therapy. In our study, we used serum-free medium, since our preliminary experiments demonstrated that the quantity of GMSC-derived EVs increased under serum deprivation compared with the exosome-depleted fetal bovine serum (FBS)-containing culture conditions, which presents a clinical advantage. In general, starvation stress can cause cells to activate survival pathways and secrete factors that counteract severe conditions. While serum starvation has the potential to influence exosomal biology^31^, previous studies have demonstrated that serum-free conditions can enhance the therapeutic efficiency of MSCs^32,33^. In this context, in addition to reducing the possibility of introducing bovine-derived artifacts^34^, approximately 45% of the published papers used serum-free medium for isolation of MSC-EVs^35^. Considering these evidences, we used serum-free medium as the optimal culture condition in this study.

In the present study, we reported that dual TNF-α/IFN-α priming in GMSCs synergistically augmented EVs-induced M2 macrophage polarization through the upregulation of CD73 and CD5L. Previous studies have shown that the inflammatory environment modulates the immunoregulatory capacity of MSCs^36^, and the effects of priming MSCs with pro-inflammatory cytokines, such as TNF-α, IFN-γ, IL-1β, and LPS have been reported^37^. Among the few studies that validated the effect of combined cytokine priming on the immunoregulatory properties of MSCs, representative priming with TNF-α and IFN-γ improved their immunomodulatory functions^38^. Meanwhile, studies on other cytokine combinations have recently reported a synergistic effect of TNF-α/IL-10 on the secretion of PGE2^39^ and IFN-γ/IL-1β on the expression of PGE2 and IDO^40^, which promotes the immunosuppressive capacity of MSCs. However, owing to the complex cross-talk between cytokine signaling, detailed downstream molecular signaling has not been elucidated. In our previous study, we demonstrated that TNF-α stimulation enhanced exosomal CD73 expression in GMSCs, which is essential for M2 macrophage induction^16^. In this study, we observed that the dual stimulation of GMSCs with TNF-α and IFN-α synergistically increased exosomal CD73 expression in EVs (Fig. 1). The expression of CD73 is upregulated at the site of inflammation and is increased by INF-α in endothelial cells^24^. However, it is also simultaneously activated by TNF-α^41^. Both of these are consistent with our current data. We also observed that enhanced CD73 expression was caused by the upregulation and nuclear translocation of HIF-1α (Fig. 2). Recent studies have revealed the transcription factors regulating CD73 expression^42^, and CD73 has a HIF-1 binding DNA-consensus motif in the promoter region. Importantly, HIF-1α binds directly to the CD73 promoter, thereby, activating CD73 expression^43^ and is also induced by both TNF-α^44^ and IFN-α^45^. Consistent with these reports, our current study demonstrated enhanced expression of CD73 by HIF-1α overexpression plasmid (Fig. 3) and inhibition of CD73 expression by HIF-1α siRNA under TNF-α/IFN-α stimulation (Fig. 4) in both GMSCs and GMSC-derived EVs. Furthermore, signal transduction between mTOR-dependent induction of HIF-1 activity^46^ and TNF-α/IFN-α-mediated mTOR activation^47,48^ was confirmed by rapamycin treatment of GMSCs (Fig. 4d), indicating that the mTOR-HIF-1α-CD73 axis is essential for TNF-α/IFN-α-induced CD73 expression in GMSCs. In the current study, we examined the effect of EVs on macrophage polarization after M1 macrophage induction (Fig. 5a). Accordingly, we further confirmed that EVs-TNF/IFN exhibited superior ability to switch M1 macrophages into the M2 phenotype (Fig. 5). It is widely accepted that HIF-1α is a central transcription factor in the hypoxia response, and hypoxic preconditioning of MSCs is one of the main MSC priming strategies^49^. Therefore, the involvement of HIF-1α in TNF-α/IFN-α priming is reasonable, and further application of GMSC-derived EVs should be validated in future studies.

We next investigated the possibility of another molecular mechanism that reinforces EVs-TNF/IFN–mediated M2 macrophage polarization. While attempting to identify any exosomal protein that modulates inflammatory responses independently of CD73, we found interesting studies reporting a novel role of CD5L as a driver of M2 macrophage polarization^29^, and CD5L was identified as a new cell surface marker in hBM-MSCs^28^. CD5L is a soluble protein belonging to the scavenger receptor cysteine-rich superfamily, and is mainly secreted from macrophages^50^. While CD5L is involved in the pathogenesis of the inflammatory processes to that control lipid metabolism by inhibiting the apoptosis of macrophages, it also promotes an anti-inflammatory cytokine profile in response to TLR activation^51^. Recent studies have revealed that CD5L is secreted from the olfactory mucosa MSC^52^. It is also compiled in the EV-specific database, ExoCarta^53^. However, the effect of exosomal CD5L on macrophage phenotype remains unknown. These reports prompted us to investigate the possible involvement of CD5L in EVs-TNF/IFN–mediated M2 macrophage polarization. In the current study, we observed a significant upregulation of CD5L mRNA expression by the combined stimulation of TNF-α and IFN-α in GMSCs (Fig. 4). Our data showed that TNF-α/IFN-α stimulation dramatically enhanced exosomal CD5L expression with a slight increase in the secreted CD5L protein (< 10 pg/mL), indicating that TNF-α and IFNα-induced expression of CD5L proteins was mostly released in the form of CD5L-overexpressed EVs-TNF/IFN of GMSCs. The expression of CD5L is positively regulated by two transcription factors, ID3^29^ and LXR^54^, which regulate the inflammatory responses by inhibiting inflammatory cytokines^55^. Accordingly, qRT-PCR analysis revealed that combined stimulation with TNF-α and INF-α initially upregulated ID3 mRNA expression within 6 h and then enhanced LXR mRNA expression at 24 h (Fig. 6). Altogether, our data suggest that this time lag between the induction of these transcription factors contributes to the continuous expression of CD5L in GMSCs. After confirmation that CD5L has the ability to induce M2 macrophage polarization (Fig. 7), knockdown of CD5L in GMSCs was performed. This inhibited EVs-mediated M2 macrophage marker expression (Fig. 8), demonstrating that exosomal CD5L was essential for TNF-α/IFN-α induced synergistic effects on M2 macrophage induction.

**Figure 9:**
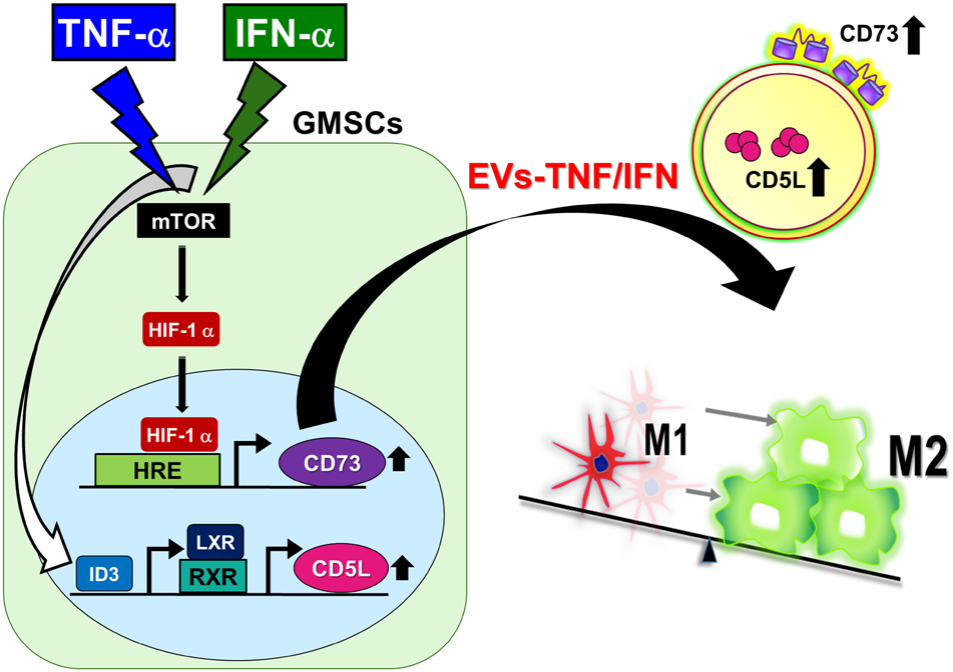
Proposed mechanism for enhancing EVs-mediated M2 macrophage polarization by dual TNF-α/IFN-α preconditioning in GMSCs. EVs from GMSCs stimulated with TNF-α and IFN-α synergistically promote M2 macrophage polarization by upregulating exosomal CD73 and CD5L. Combined priming of TNF-α and IFN-α enhances the expression of CD73 through activation of the HIF-1α-mTOR pathway, and the upregulation of CD5L was transcriptionally mediated by LXR and ID3 in GMSCs.

In conclusion, our study demonstrated that EVs from GMSCs stimulated with the combination of TNF-α and IFN-α promote M2 macrophage polarization by the upregulation of exosomal CD73 and CD5L. Compared with stimulation with a single cytokine, the combination of TNF-α and IFN-α synergistically enhanced the expression of CD73 through the activation of the HIF-1α-mTOR pathway and the upregulation of CD5L was transcriptionally mediated by LXR and ID3 in GMSCs. These findings based on the molecular mechanisms are summarized in Fig. 9. Considering the impact of macrophage plasticity on disease^56^, GMSCs-derived EVs preconditioned by TNF-α and IFN-α could be a promising therapeutic tool for the treatment of inflammatory diseases.

## Materials and methods

### Ethics statement and preparation of human tissue samples

All human gingival tissues were obtained as discarded clinical samples using the approved institutional review board (IRB) protocol at Kyushu University Hospital. The procedures for using human samples were conducted in accordance with the Declaration of Helsinki and were approved by the Kyushu University Institutional Review Board for Human Genome/Gene Research (Protocol Number: 2019-374). Written informed consent was obtained from all the subjects. Human gingival tissues were obtained from three 27–32 years old adult donors who were early stages of periodontitis. After initial treatment for periodontitis, gingivectomy was performed to eliminate the distal pockets adjacent to the terminal molars. The inclusion and exclusion criteria of subjects were listed in Supplementary Table 1.

### Cytokines and reagents

Recombinant human TNF-α, IFN-α, IFN-γ, IL-4, IL-13, and macrophage-colony stimulating factor (M-CSF) were purchased from BioLegend (San Diego, CA, USA) and recombinant human CD5L was purchased from R&D Systems (Minneapolis, MN, USA). LPS from *Escherichia coli* 055:B5 was obtained from Sigma-Aldrich (St. Louis, MO, USA). Rapamycin was purchased from Wako Pure Chemical Industries Ltd. (Osaka, Japan).

### Cell culture

Human GMSCs were isolated and cultured as reported in our previous studies^16,20,57,58^. Briefly, gingival tissues were gently separated, minced, and digested with phosphate-buffered saline (PBS) solution containing collagenase type I (2 mg/mL) (Worthington Biochemicals, Lakewood, NJ, USA) and dispase II (4 mg/mL) (Sanko Junyaku, Tokyo, Japan) for 1 h at 37 °C. Single-cell suspensions from the gingiva were obtained by passing the culture through a 70-μm strainer (Falcon, BD Biosciences Discovery Labware, Bedford, MA, USA). All nucleated cells were seeded in 100-mm culture dishes with complete media containing alpha modification of Eagle’s medium (Invitrogen, Waltham, MA, USA) supplemented with 10% fetal bovine serum (FBS, Hyclone Laboratories, USA), 2 mM l-glutamine (Invitrogen, Carlsbad, CA, USA), 10 mM l-ascorbic acid phosphate (FUJIFILM Wako, Japan), and 100 U/mL penicillin/streptomycin (Gibco Life Technologies, country), followed by an initial incubation for 48 h at 37 °C in 5% CO_2_ and 95% humidity. The cultures were subsequently washed twice with PBS to eliminate non-adherent cells. The attached cells were further cultured for another 12 days under the same conditions in the complete medium mentioned above, and cells at the 3rd–5th passages were used for subsequent experiments. Human CD14^+^ peripheral blood-derived monocytes (PBMCs; lot no. 2050813521) were purchased from Lonza (Basel, Switzerland). Cells were seeded in 6-well plates (2 × 10^5^ cells/well) and cultured in RPMI 1640 medium (Nacalai Tesque, Kyoto, Japan) supplemented with 10% heat-inactivated FBS, 2 mM glutamine (Nacalai Tesque), 1% sodium pyruvate (Nacalai Tesque), 1% nonessential amino acids (Nacalai Tesque), and 25 ng/mL of M-CSF. To generate macrophages, monocytes were cultured for 7 days with a single change of fresh medium on day 3–4 after the initiation of the culture. To polarize M1 or M2 macrophages, macrophages were further treated with 10 ng/mL LPS, 20 ng/mL IFN-γ or 20 ng/mL IL-4, and 20 ng/mL IL-13 for 24 h.

### Flow cytometry

The expression of CD73 in GMSCs and CD11b, CD206, and CD86 in PBMC-derived macrophages was analyzed using FACS Calibur (Becton Dickinson, Franklin Lakes, NJ, USA) and CellQuest software (Becton Dickinson). The adherent cells were washed with PBS, collected using Accutase (Nacalai Tesque), and resuspended in 50 μL staining buffer (BD Pharmingen, San Diego, USA). The harvested cells were blocked with 2 μL Human Trustain FcX (Fc receptor Blocking Solution; BioLegend) for 10 min at 25 °C and stained with 2 μL antibodies: FITC anti-human CD73, Alexa Fluor® 488 anti-human CD11b, PE anti-human CD206, APC anti-human CD206, PE anti-human CD86, and APC anti-human CD86 (BioLegend, San Diego, CA USA) in the dark for 45 min at 4 °C.

### Isolation and characterization of EVs

EVs were prepared according to the protocol reported in our previous study^16^ and the recommendations of the International Society of Extracellular Vesicles^59^. For preconditioning of GMSCs, cells in 100-mm culture dishes were grown to achieve a confluence of 70–80%. After the medium was aspirated, cells were rinsed three times with PBS and treated with 100 ng/mL of recombinant human TNF-α and IFN-α, and 50 ng/mL of TNF-α and IFN-α in a serum-free medium and incubated for 48 h prior to supernatant harvesting. Controls are maintained in a serum-free medium without any additions. GMSC-derived EVs were isolated from serum-free conditioned media using the MagCapture™ Exosome Isolation Kit PS (FUJIFILM Wako)^60^. Cells were preconditioned in a serum-free medium for 48 h. Medium was then collected and centrifuged at 10,000×*g* for 30 min to eliminate other large extracellular vesicles. The cleared supernatants were passed through 0.22 mm filter membranes and concentrated using a Vivaspin-20 concentrator (Sartorius, Göttingen, Germany). The amount of protein in EVs was quantified using a BCA Protein Assay Kit (Takara Bio, Otsu, Japan), and 2.5 mg/mL of EVs were used for the stimulation of macrophage. Transmission electron microscopy (TEM) analyses were performed by the Hanaichi Ultrastructure Research Institute (Okazaki, Japan). The Nanoparticle Characterization System (NanoSight, Malvern Instruments, UK) was used to measure size distribution of EVs.

### Confocal microscopy

GMSCs (3 × 10^4^ cells/well) were seeded onto glass coverslips and stimulated with the indicated amounts of TNF-α and IFN-α for 6 and 24 h to observe the nuclear accumulation of HIF-1α. The cells were washed twice with PBS (pH 7.4), fixed with 4% paraformaldehyde for 20 min, blocked with Blocking One Histo (Nacalai Tesque) for 5 min at room temperature, treated with 0.5% Triton X-100 (Junsei, Tokyo, Japan), which penetrated into the cells, for 10 min at room temperature, and stained with primary anti-HIF-1α (1:200, D1S7W, Cell Signaling Technology) antibody for 1 h at room temperature. Immunofluorescent labelling of HIF-1α was performed using anti-HIF-1α (1:200, D1S7W, Cell Signaling Technology). Subsequently, Alexa Fluor® 488 secondary antibody (1:1000; Life Technologies) was used as secondary antibody for 1 h in the dark at room temperature. Nuclei were stained using SlowFade® Diamond Antifade Mountant with 4,6-diamidino-2-phenylindole (DAPI) (Life Technologies, Waltham, MA, USA). Images were captured using a confocal laser-scanning microscope (Carl Zeiss LSM 700; Oberkochen, Germany) and ZEN 2012 software.

### Quantitative real-time PCR (qRT-PCR) analysis

qRT-PCR was performed as previously described^16^. Total RNA was isolated from cells using ISOGEN II (Nippon Gene, Tokyo, Japan), and first-strand cDNA was synthesized using PrimeScript RT Master Mix (Takara Bio, Otsu, Japan). qRT-PCR was performed using the KAPA SYBR® FAST qPCR Kit Master Mix (Kapa Biosystems, Woburn, MA, USA) on a StepOnePlus™ Real-Time System (Applied Biosystems, Carlsbad, CA, USA) under the following conditions: 95 °C for 3 min, 40 cycles of 95 °C for 3 s, and 60 °C for 30 s. To correct varying copies of first-strand cDNA templates, a passive reference dye (Rox) was added to the PCR master mix. The results were recorded and analyzed using StepOne™ software V2.2.2 (Applied Biosystems, Life Technologies Corporation) utilizing the auto-calculated threshold cycle. The ΔΔC_T_ method was used to calculate the relative expression levels of the individual genes, and glyceraldehyde-3-phosphate dehydrogenase (*GAPDH*) was used as an internal control. Primer sequences used in this study are listed in Supplementary Table 2.

### Western blot analysis

Western blot was performed as previously described^16^. Cells were washed with PBS and lysed in Cytobuster Protein Extraction Reagent (Novagen Inc., Madison, Wisconsin, USA) supplemented with a protease inhibitor cocktail (Nacalai Tesque). Protein samples were separated on polyacrylamide gels and transferred to polyvinylidene difluoride membranes. The membranes were incubated with the following primary antibodies: anti-CD9 (1:1000, Ts9, Thermo Fisher Scientific), anti-NT5E/CD73 (1:1000, D7F9A, Cell Signaling Technology), anti-CD63 (1:1000, Ts63, Thermo Fisher Scientific), anti-CD81 (1:1000, Ts81, Thermo Fisher Scientific), anti-HIF-1α (1:1000, D1S7W, Cell Signaling Technology), anti-mTOR (1:1000, 7C10, Cell Signaling Technology), anti-p-mTOR (1:1000, S2448, Cell Signaling Technology), anti-CD5L/CD-2 (1:1000, ab45408, Abcam), and anti-β-actin (1:1000, 13E5, Cell Signaling Technology). Blotted membranes were visualized using a densitometry technique on a LAS-4000 mini luminescent image analyzer (GE Healthcare, Marlborough, MA, USA) and quantified using Multi Gauge 3.1 software (FUJIFILM, Tokyo, Japan). For reference, all full-length blots are presented in Supplementary Materials.

### Enzyme-linked immunosorbent assay

Cell supernatants were collected, and the expression levels of CD5L were measured using a Human CD5L ELISA Kit (Thermo Fisher Scientific), according to the manufacturer’s instructions. The plates were read using a microplate reader at 450 nm with a background correction at 630 nm.

### Cell transfection

GMSCs were seeded in 6-well plates (5 × 10^5^ cells/well) a day before transfection. At approximately 60–70% confluence, the cells were transfected with indicated amounts of the expression plasmids using 2 µL of Lipofectamine 3000 (Thermo Fisher Scientific), according to manufacturer's instructions. For the control, cells were transfected with empty vectors under the same conditions. Human HIF-1α expression plasmid (pCAG-HIF1 alpha) and control plasmid (pCAGEN) were obtained from Addgene (http://www.addgene.org/). Transfection of siRNAs was carried out using Lipofectamine™ RNAiMAX Transfection Reagent (Thermo Fisher Scientific) for 24 h according to our reverse transfection protocol^61^. Stealth™ RNAi duplexes, comprising mixtures of three different siRNAs against human CD73 (HSS107326, HSS107328, and HSS181585), and HIF-1α (HSS179231, HSS104774, and HSS104775) were obtained from the Invitrogen Corporation (Invitrogen Life Technologies, Carlsbad, CA, USA). Stealth™ RNAi negative control duplex (Medium GC Duplex, Invitrogen Life Technologies, Carlsbad, CA, USA) was used as the control.

### Statistical analysis

Data were analyzed using the JMP Pro 16 software (SAS Institute Inc., Cary NC, USA). Comparisons between two groups were performed using an independent unpaired two-tailed Student’s *t-*test, and comparisons between more than two groups were performed using one-way analysis of variance (ANOVA) with Bonferroni correction. Statistical significance was set at *P* < 0.05.

## Supplementary Information

Supplementary Information 1.

Supplementary Tables.

## Data Availability

The data used to support the findings of this study are available from the corresponding author on request.
