# Supplementary material for: Extracellular vesicles derived from GMSCs stimulated with TNF-α and IFN-α promote M2 macrophage polarization via enhanced CD73 and CD5L expression
Source: Sci Rep. 2022 Aug 3;12:13344. doi: 10.1038/s41598-022-17692-0 (PMC9349189; doi:10.1038/s41598-022-17692-0)
Supplement: Supplementary file 2 — Supplementary Tables. [file 41598_2022_17692_MOESM2_ESM.docx]

**Supplementary Tables for**

**Extracellular vesicles derived from GMSCs stimulated with TNF-α and IFN-α promote M2 macrophage polarization via enhanced CD73 and CD5L expression**

Yukari Watanabe^1^, Takao Fukuda^1*^, Chikako Hayashi^1^, Yuki Nakao^1^, Masaaki Toyoda^1^, Kentaro Kawakami^1^, Takanori Shinjo^1^, Misaki Iwashita, Hiroaki Yamato^1^, Karen Yotsumoto^1^, Takaharu Taketomi^2^, Takeshi Uchiumi^3^, Terukazu Sanui^1**^ and Fusanori Nishimura^1^

^1^ Department of Periodontology, Division of Oral Rehabilitation, Faculty of Dental Science, Kyushu University, Fukuoka, Japan

^2^ Department of Dental and Oral Surgery, St. Mary’s Hospital, Fukuoka, Japan

^3^ Department of Clinical Chemistry and Laboratory Medicine, Graduate School of Medical Sciences, Kyushu University, Fukuoka, Japan

*Corresponding author: Takao Fukuda, D.D.S., PhD, lecturer

**Corresponding author: Terukazu Sanui, D.D.S., PhD, lecturer

Department of Periodontology, Division of Oral Rehabilitation, Faculty of Dental Science, Kyushu University, 3-1-1 Maidashi, Higashi-ku, Fukuoka 812-8582, Japan

Tel:+81-92-642-6358, Fax: +81-92-642-6360

E-mail: tfukuda@dent.kyushu-u.ac.jp (T. Fukuda)

sanuteru@dent.kyushu-u.ac.jp (T. Sanui)

**Supplementary Table 1.**

Criteria for selecting subjects

| 1. Those who were diagnosed as having chronic periodontitis and indicated for periodontal surgery |
| --- |
| 1. Those who understand the purpose of this study and providing written informed consent. |
| 1. Patients not younger than 20 years old at the time of informed consent. |

Criteria for excluding subjects

| 1. Patients with anaemia (Hb < 8g/dL) |
| --- |
| 1. Patients in poor general condition (PS ≥ 3) |
| 1. Others who the investigators determined as unsuitable for this observational study |

**Supplementary Table 2.**

Primer sequence used for quantitative RT-PCR

| **Gene** | **Forward primer** | **Reverse primer** |
| --- | --- | --- |
| ***human CD73*** | 5'-**GCCTGGGAGCTTACGATTTTG**-3' | 5'-**TAGTGCCCTGGTACTGGTCG**-3' |
| ***human HIF-1α*** | 5'-**CATCTCCATCTCCTACCCACAT** -3' | 5'- **ACTCCTTTTCCTGCTCTGTTTG** -3' |
| ***human CD5L*** | 5'-**GACGAGAAGCAACCCTTCAG** -3' | 5'-**CCCAGAGCAGAGGTTGTCTC** -3' |
| ***human* ID3** | 5'-**TCATCTCCAACGACAAAAGG** -3' | 5'-**ACCAGGTTTAGTCTCCAGGAA** -3' |
| ***human* LXR** | 5'-***ATCCCCATGACCGACTGATGT***-3' | 5'-***TGCAGACGCAGTGCAAACA***-3' |
| ***human GAPDH*** | 5'-**ATCAAGAAGGTGGTGAAGCAGG**-3' | 5'-**GTCATACCAGGAAATGAGC**-3' |
